# Accelerated Formation of 2D Ruddlesden—Popper Perovskite Thin Films by Lewis Bases for High Efficiency Solar Cell Applications

**DOI:** 10.3390/nano12111816

**Published:** 2022-05-26

**Authors:** Swathi M. Gowdru, Jou-Chun Lin, Szu-Tan Wang, Yi-Chia Chen, Kuan-Chang Wu, Cheng-Nan Jiang, Yu-Dian Chen, Shao-Sian Li, Yuan Jay Chang, Di-Yan Wang

**Affiliations:** 1 Department of Chemistry, Tunghai University, Taichung 40704, Taiwan; swathimb3@gmail.com (S.M.G.); jouchun1908@thu.edu.tw (J.-C.L.); ybbis1124@yahoo.com.tw (S.-T.W.); yichia899@gmail.com (Y.-C.C.); leo19981009leo@gmail.com (K.-C.W.); jiangjengnan@gmail.com (C.-N.J.); 510087d@gmail.com (Y.-D.C.); jaychang@thu.edu.tw (Y.J.C.); 2 Department of Materials and Mineral Resources Engineering, Institute of Materials Science and Engineering, National Taipei University of Technology, Taipei 10608, Taiwan

**Keywords:** 2D Ruddlesden—Popper Perovskite, BA_2_(MA)_n−1_Pb_n_I_3n+1_, in-situ X-ray diffraction, grazing-incidence small-angle X-ray scattering, PL mapping

## Abstract

Various types of 2D organic–inorganic perovskite solar cells have been developed and investigated due to better electron transport behavior and environmental stability. Controlling the formation of phases in the 2D perovskite films has been considered to play an important role in influencing the stability of perovskite materials and their performance in optoelectronic applications. In this work, Lewis base urea was used as an effective additive for the formation of 2D Ruddlesden—Popper (RP) perovskite (BA)_2_(MA)_n−1_Pb_n_I_3n+1_ thin film with mixed phases (n = 2~4). The detailed structural morphology of the 2D perovskite thin film was investigated by in situ X-ray diffraction (XRD), grazing-incidence small-angle X-ray scattering (GISAXS) and photoluminescence mapping. The results indicated that the urea additive could facilitate the formation of 2D RP perovskite thin film with larger grain size and high crystallinity. The 2D RP perovskite thin films for solar cells exhibited a power conversion efficiency (PCE) of 7.9% under AM 1.5G illumination at 100 mW/cm^2^.

## 1. Introduction

Organic–inorganic two-dimensional (2D) Ruddlesden—Popper (RP) perovskites have gained tremendous research interest as a promising alternative to three-dimensional (3D) perovskites due to their excellent photovoltaic properties. In particular, organic–inorganic 2D RP perovskites have high charge carrier mobility, high light absorption coefficient, higher exciton binding energy and reasonable band gap [1,2,3,4]. Recently, organic–inorganic 2D RP perovskites have been widely used to achieve remarkable results in all kinds of optoelectronic devices such as light-emitting diodes [2,5,6], solar cells [1,4,7,8] and optical lasers [9,10]. In addition, the synthesis of 2D thin films with phase purity (having only one n value) along with high n values (n = 2, to 5) was accomplished, which led to the creation of high-efficiency thin film solar cells based on 2D RP perovskites [11,12,13]. These 2D RP perovskites materials have the general chemical formula (BA)_2_(MA)_n__−__1_Pb_n_I_3n+1_ where BA and MA represent the butylammonium cation [C_4_H_9_NH_3_]^+^ and the methylammonium cation [CH_3_NH_3_]^+^, respectively. The growth method of 2D RP perovskites with a large single crystal was obtained by slow evaporation with a constant-temperature solution-growth technique. To form a solid thin film of 2D RP perovskites, the role of the solvent annealing with adjusting precursor−solvent interaction played an important role in phase purity, grain size and surface quality for further optoelectronic applications. Therefore, the development of fast and efficient methods for the fabrication of 2D RP perovskite thin films is key to future large-scale industrial applications.

In recent reports, the additive method has been employed to improve the performance of perovskites solar cells (PSCs), where the organic–inorganic 3D PSCs have surpassed PCE of 25%, forthcoming the performance of crystalline silicon solar cells [14,15,16]. However, stability issues still prevent the large-scale industrialization of 3D PSCs. Adding additives to the perovskites can stabilize PSCs and thus improve their performance. Previous reports have also demonstrated that doping perovskites with polymers, fullerenes and pyridines can enhance the formation of PSCs [17,18,19]. The addition of additives can also promote crystallization, surface passivation and ion regeneration to generate a stable 2D PSCs [20]. In addition, dimethyl sulfoxide (DMSO) volatile solvent and urea/thiourea as suitable additives have been used for the fabrication of 3D perovskites MAPbI_3_ solar cells with PCE 18%, and other multiple cation-lead halide perovskites devices, with PCE exceeding 20% [21,22,23,24]. For example, Yang et al. reported MAPbI_3_-derived normal (ITO/TiO_2_) PSCs achieved an efficiency of 18.25% with the addition of urea and thiourea as additives and an annealing temperature of 100 °C [24]. Chuang et al. used 1% urea as an additive for MAPbI_3_-based PSCs using an extremely low annealing temperature of 85 °C and accomplished PCE of 18.8% [25]. The urea additive was found to improve the trap passivation in perovskites grain boundaries, resulting in reducing the occurrence of trap defects and recombination and then enhancing the carrier lifetime. Therefore, it is still an important subject to study the role of urea molecules in the formation mechanism of 2D RP perovskite thin films for further applications.

In this study, we report a PCE of 7.9% under AM 1.5 G illumination for 2D RP perovskite thin films (BA)_2_(MA)_n−1_Pb_n_I_3n+1_ with mixed phases (n = 2, 3 and 4), modified by Lewis base urea molecules as an effective additive to the precursor. The additive urea can help accelerate the formation of a 2D RP perovskite thin film with large grain size and high crystallinity by using a facile spin-casting process. Furthermore, the detailed structural morphology of a 2D RP perovskite thin film was characterized by in situ X-ray diffraction (XRD), grazing-incidence small-angle X-ray scattering (GISAXS) and photoluminescence (PL) mapping.

## 2. Results and Discussion

**Figure 1:**
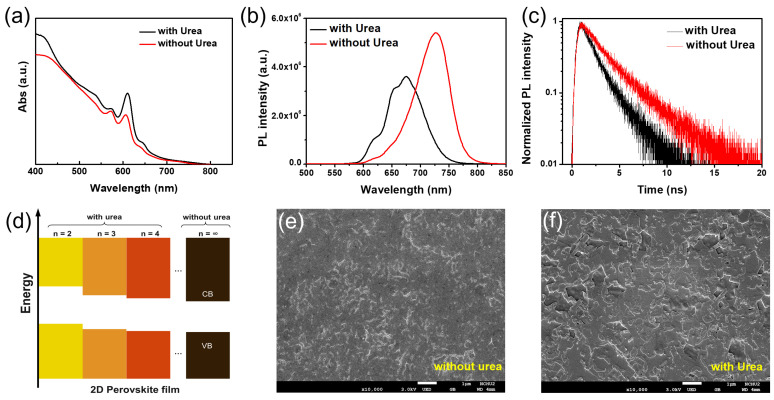
(**a**) The absorption spectra, (**b**) PL spectra and (**c**) TRPL spectra of pristine thin film (without urea) and that with urea treatment. (**d**) Comparative bandgap energy alignment of (BA)_2_(MA)_n__−__1_Pb_n_I_3__n__+1_ perovskites with and without urea treatment. The SEM images of (**e**) pristine thin film (without urea) and (**f**) that with urea treatment.

To fabricate the 2D RP perovskite film with mixed phases, we used a simple and facile method by the spin-casting process, annealing at 90 °C. A precursor of urea-treated (BA)_2_(MA)_2_Pb_3_I_10_ solution were fabricated from the optimized reaction between PbI_2_, methylammonium iodide (CH_3_NH_3_I), and butylammonium iodide (C_4_H_9_NH_3_I) in hydrogen iodide solution with additionally of 5 mg of urea. The preparation method was modified by a previous report [26]. The optical property and structure morphology of pristine thin film (without urea treatment) and that with urea treatment were obtained and the results are shown in Figure 1. The absorption spectra of both films are observed in Figure 1a. The results indicate that there are several exciton peaks found at ca. 572, 609, and 646 nm corresponding to n value of 2, 3 and 4, which are more prominent for urea-treated films in contrast to the pristine film [27]. The related schematic illustration of the chemical structure of (BA)_2_(MA)_n−1_ Pb_n_I_3n+1_ 2D perovskite unit cell with a different n value is provided in the Figure S1. Figure 1b illustrates the PL spectra for two films. The results show that the corresponding PL peaks of pristine thin film are located at 725 nm, but that of urea treated film exhibits a blue shift to 675 nm, along with two shoulder peaks at 653 nm and 616 nm, which are matched to the exciton peaks found in the absorption spectra. To investigate the carrier behavior of two films on indium tin oxide (ITO) substrate, time-resolved photoluminescence (TRPL) spectroscopy was performed, and the result is shown in Figure 1c. The results show that TRPL demonstrates a lifetime (τ_ave_) of 3.39 ns and 1.98 ns for the pristine and urea treated films, respectively. The corresponding energy levels of (BA)_2_(MA)_n−1_ Pb_n_I_3n+1_ 2D perovskite with and without urea treatment were obtained in Figure 1d. The energy gap of 2D perovskite film with urea treatment are 2.15 eV (n = 2), 2.06 eV (n = 3) and 1.95 eV (n = 4) and that without urea treatment are 1.7 eV (n ≈ ∞). The energy gap was calculated from multiple absorption peaks in the absorption spectra. [27] The band position of valence band and conduction band of 2D perovskites with n = 2 to 4 were referred to the exciton peaks in the absorption spectra. Figure 1e,f show the SEM images of the pristine film and urea treated film, respectively. We found that the pristine film exhibited small grain size and poor surface coverage with pores, but the urea treated film demonstrated larger grain size with continuous grain boundaries, and almost no pores were found on the surface of the thin films. The overall results indicate that with urea treatment, the formation of 2D RP film with mixed phases (n = 2 to 4) will be facilitated successfully in comparison with that without urea treatment.

**Figure 2:**
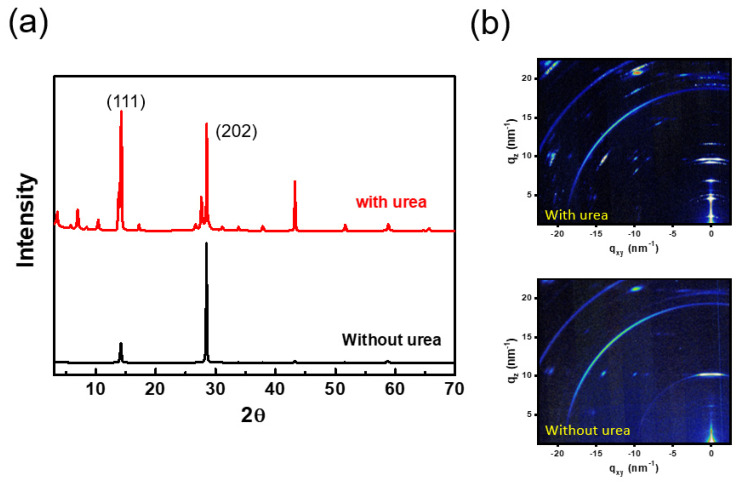
(**a**) XRD features of the pristine film and urea treated film. (**b**) 2D GISAXS patterns of the pristine film and urea treated film.

Figure 2a demonstrates the XRD features of the pristine film and urea treated film. Two films show two dominant (111) and (202) faceted peaks at 2θ = 14.1° and 28.5° with lattice distance of 6.35 and 3.12 Å, respectively. The results represent that the crystal domain shows a bulk orientation in these two films. Furthermore, the crystallite size was calculated by the Scherrer equation, and the average crystallite size of with/without urea treatment samples was calculated as 42.1 nm and 39.4 nm, respectively. The results indicated that the larger crystallite size of 2D perovskite materials with urea treatment were obtained after urea treatment. Moreover, there are several diffraction peaks found in the lower 2θ degree (2θ < 13°) for urea treated film. However, no peak at this range was found in the pristine film. The results indicate that the urea treated film exhibits a formation of 2D RP phase with lower-n values. To further confirm this result, the characterization of GISAXS measurement (National Synchrotron Radiation Research Center, Hsinchu city, Taiwan) was obtained and the result is shown in Figure 2b. The GISAXS patterns are shown for the pristine film and urea treated film. The same dominant Bragg diffraction spots at q = 10.0 nm^−1^ were found in two films. Remarkably, the urea treated films show additional and distinct spots in the q range of 0~10 nm^−1^, which corresponds to 2D mixed phases with n value of ~2, 3 and 4. According to these characterizations, the additive urea molecules played an important role in the phase formation of 2D RP perovskites by a spin casting process.

**Figure 3:**
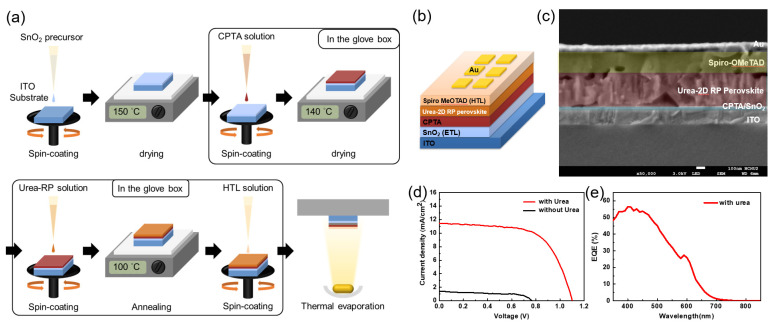
(**a**) The schematic illustration of device fabrication process and (**b**) the resulting device of the urea treated 2D RP perovskite solar cell (PSC) with the architecture of indium tin oxide (ITO)/SnO_2_/CPTA/urea treated 2D RP perovskite/ Spiro-OMeTAD/Au. (**c**) A cross-sectional SEM image of the urea treated 2D RP PSC device. (**d**) J–V curves of 2D RP PSCs without and with urea treatment under simulated AM 1.5G illumination (100 mW/cm^2^). (**e**) The EQE spectrum of 2D RP PSCs with urea treatment.

The fabrication process and the device of urea treated 2D RP perovskite solar cell (PSC) with the architecture of indium tin oxide (ITO)/SnO_2_/CPTA/urea treated 2D RP perovskite/ Spiro-OMeTAD/Au is shown in Figure 3a,b, respectively. Figure 3c shows a cross-sectional scanning electron microscopy (SEM) (Tunghai university, Taichung city, Taiwan) image of the urea treated 2D RP PSC device. The 2D perovskite absorber has a thickness of about 350 nm with conformal flat coverage on ITO/SnO_2_/CPTA. Figure 3d shows J–V curves of 2D RP PSCs without and with urea treatment under simulated AM 1.5G illumination (100 mW/cm^2^). The detailed J–V values are summarized in Table 1. These 2D RP PSCs with urea treatment exhibit better performance with a Voc of 1.10 V and FF of ~62.8% than that without urea treatment (Voc~0.77 V, FF~59.0%). Furthermore, the 2D RP PSC with urea treatment yielded a PCE of 7.9% with a short-circuit current density (Jsc) of 11.4 mA/cm^2^. However, the PSC without urea treatment only exhibited a PCE of 0.64% with a Jsc of 1.4 mA/cm^2^. Figure 3e showed the EQE spectrum of 2D RP perovskite solar cell (PSC) with urea treatment. The result indicated that The PSC with urea treatment exhibited a broad EQE spectrum from 350 to 700 nm, with a significantly high value of >55% at a wavelength range from 400 to 500 nm, which gave an integrated photocurrent of 11.18 mA/cm^2^. This value is consistent with the JSC value under simulated AM 1.5G irradiation. The overall results indicate that the performance of urea treated 2D RP PSC can be improved through the successful formation of larger grain size and continuous boundary in a 2D RP perovskite thin film.

**Figure 4:**
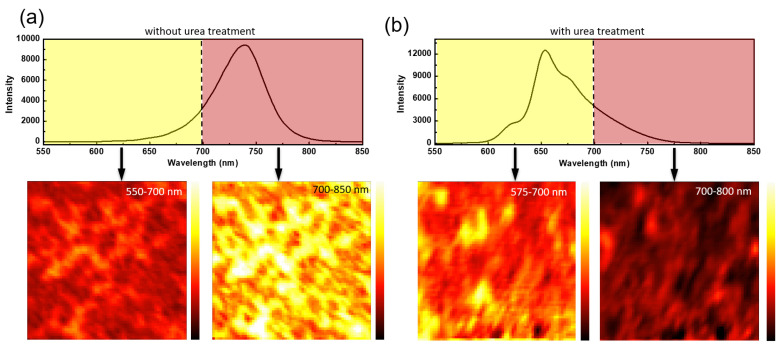
2D intensity mapping of the PL spectra for a 5 µm × 5 µm area of (**a**) the pristine 2D RP film and (**b**) the urea treated film. The two wavelength ranges (one is 550 nm to 700 nm and the other is 700 nm to 850 nm) of PL mapping were selected.

Figure 4 shows 2D intensity mapping of the PL spectra for a 5 µm × 5 µm area of the pristine 2D RP film and the urea treated film that was performed under a confocal microscope with a spatial resolution of about 500 nm. Excited by a 532 nm laser, the integrated intensity of PL mapping was collected by an Andor spectrometer with a photomultiplier tube. The two kinds of wavelength ranges (one is 550 nm to 700 nm and the other is 700 nm to 850 nm) of PL mapping were selected. The results represent that the pristine film (Figure 4a) shows a stronger PL intensity at 700 nm to 850 nm and less PL intensity contribution at 550 nm to 700 nm. Instead, the urea treated film (Figure 4b) shows the dominant distribution of PL intensity at 550 nm to 700 nm at an area of 5 µm × 5 µm. According to PL mapping, we found that with urea treatment, the 2D RP film with mixed n values formed successfully over a large area compared to the pristine films, which tended to form 3D phases.

**Figure 5:**
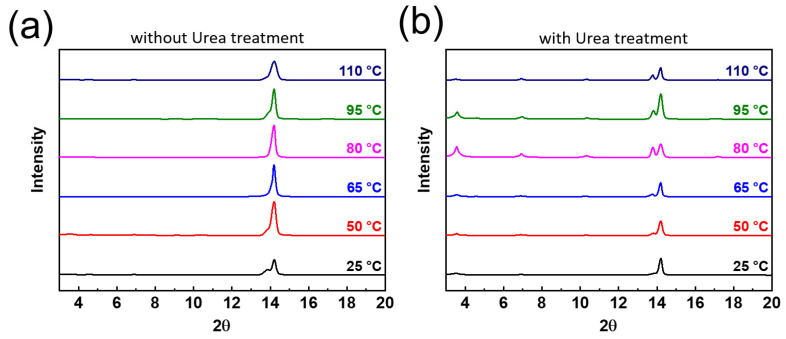
In situ XRD spectra were obtain to collect the structural information of the perovskite films (**a**) without and (**b**) with urea treatment under continuous annealing procedure from 25 °C to 110 °C.

To investigate the formation mechanism of the 2D RP perovskite thin film, in situ XRD spectra (National Synchrotron Radiation Research Center, Hsinchu city, Taiwan) (Figure 5) were obtained to collect the structural information of the perovskite film under a continuous annealing procedure from 25 °C to 110 °C. The XRD patterns were collected from the range of 2θ~3° to 20°. We found that without urea treatment, only one peak at 2θ~14° was found. No additional peak was found at a lower degree (<2θ~10°). However, with urea treatment, the three periodic repetition peaks appeared at the annealing temperature of 50 °C. As the temperature increased, the peak intensity also increased. When the annealing temperature was increased to 90 °C, the strongest peak intensity was reached, which means that the 2D RP phase in the perovskite film can form the best crystallinity. In the literature report, [24] the Lewis base additives has been utilized to improve the crystallization of 3D perovskite materials due to the increase in the activation energy (Ea) of crystallization reaction. With the increase in Ea, the crystallization rate could become slower, resulting in the growth of larger grain size of 3D perovskite crystal, along with reducing grain boundaries of perovskite films to passivate the defect sites. Because the activation energy of 2D perovskite materials with a lower n value is larger than that of 3D perovskite materials with urea treatment, the formation activation energy of 2D perovskite materials was increased, resulting in a much slower crystallization rate and causing the successful formation of 2D perovskite materials with lower n values in comparison with that without urea treatment.

## 3. Conclusions

We have demonstrated that Lewis base urea successfully acts as an effective additive for the rapid formation of 2D Ruddlesden—Popper (RP) perovskite (BA)_2_(MA)_n−1_ Pb_n_I_3n+1_ thin film with mixed phases at a low temperature. Better surface and crystallinity of 2D RP perovskite films with a larger grain size were obtained by urea treatment. Highly efficient 2D RP perovskite solar cells with 7.9% PCE under AM 1.5G illumination were showcased. An improvement in PCE efficiency and reproducibility was observed due to the ability of the urea additive to modify defects and traps at grain boundaries.

## 4. Experimental Section

### 4.1. Chemicals

All the chemicals were used as received without any further purification, including Lead(II) oxide (99%, J.T.Baker, England), Hydroiodic acid (57%, Sigma-Aldrich, St. Louis, MO, USA), Hypophosphorous acid (50 wt.% in H_2_O, Acros, USA), Butylamine (BA, 99.5%, Sigma-Aldrich, USA), Methylammonium chloride (MACl, 99%, Aldrich, Belgium, WI, USA), Tin(IV) oxide (15% in H_2_O colloidal dispersion, Alfa Aesar, Ward Hill, MA, USA), C60 Pyrrolidine tris-acid (CPTA, 97%, Sigma-Aldrich, USA), Urea (99%, Sigma-Aldrich, USA), N,N-Dimethylformamide (DMF, 99.8%, Sigma-Aldrich, USA), Spiro-OMeTAD (99.8%, Lumtec, New Taipei, Taiwan), chlorobenzene (CB, 99.5%, Sigma-Aldrich, USA), 4-tert-Butylpyridine (tBP, 96%, Sigma-Aldrich, USA), Lithium bis(Trifluoromethanesulfonyl)imide (LiTHSi, 98+%, Alfa Aesar, USA), and Acetonitrile (ACN, 99.8%, Sigma-Aldrich, USA).

### 4.2. The Precursor Preparation of 2D RP Perovskite Thin Film

To prepare the precursor of 2D RP perovskite thin film, a single crystal of 2D RP perovskite materials were obtained. First, 2.232 g of PbO was dissolved in a mixture of 10 mL HI and 1.7 mL of H_3_PO_2_ and stirred at 80 °C until dissolved. In a separate flask with an ice bath containing 5ml of HI, 327 μL of BA was slowly added to and reacted for 20 min. Then, 0.45 g of MACl was added to the PbO solution and heated to 120 °C until dissolved, followed by the addition of BA solution, and then a wait of 3 min. Then, the solution was cooled to room temperature. Finally, the (BA)_2_(MA)_2_Pb_3_I_10_ crystals were isolated by suction filtration and thoroughly dried at 40 °C in a vacuum oven. For the synthesis of (BA)_2_(MA)Pb_2_I_7_, the only two differences are the amount of BA and MACl are changed to 694 μL and 0.338 g. The resulting materials were used for further fabricating the 2D RP perovskite thin film by using a spin coating process. The (BA)_2_(MA)_1_Pb_2_I_7_ crystals were also prepared by adjusting the molar ratio of the starting chemicals.

### 4.3. The Preparation of ITO Substrate

Next, 2 cm × 2 cm ITO glass substrates (Sheet resistance: 7 Ω/sq, Starek, Taipei City, Taiwan) were sonicated in soap, DI water, acetone, and IPA for 20 min sequentially. After that, the glass substrate was rinsed with deionized water and dried with pure N_2_, followed by O_2_-plasma surface treatment for 15 min.

### 4.4. Device Fabrication

A SnO_2_ precursor was prepared by adding 200 μL of SnO_2(aq)_ into 1300 μL DI water. Then, 60 μL of SnO_2_ precursor solution was spin-coated at 3000 rpm for 30 s, followed by drying for 30 min at 150 °C, and then the SnO_2_ substrates were transferred into a glovebox after being cooled to room temperature. Then, 60 μL of a 2 mg/mL solution of CPTA in DMF was spin-coated at 4000 rpm for 30 s and heated at 140 °C for 10 min. Then, 80 μL of a (BA)_2_(MA)_2_Pb_3_I_10_ solution [0.3195 g (BA)_2_(MA)_2_Pb_3_I_10_ with 5 mg of urea in 1 mL of DMF] was deposited on the ITO/SnO_2_/CPTA substrates by spin-coating at 5000 rpm for 30 s. Then, the film was annealed at 100 °C for 10 min and cooled to room temperature. A hole-transport solution was prepared by mixing 1 mL of an 80 mg/mL solution of Spiro-OMeTAD in CB, 28.5 µL of tBP, and 17.5 µL of a 520 mg/mL LiTHSi stock solution in ACN. A hole transport layer was spin-coated from the Spiro-OMeTAD solution at 2000 rpm for 30 s under N_2_ atmosphere, followed by deposition of a 100 nm patterned Au top electrode by thermal evaporation to complete the device fabrication.

### 4.5. Characterization

The absorption spectra and photoluminescence (PL) spectra were obtained by a UV/vis/NIR spectrophotometer (Jasco V-770, Tunghai university, Taichung city, Taiwan) and fluorescence spectrophotometer (HITACHI F-4500, Tunghai university, Taichung city, Taiwan), respectively. The time-resolved photoluminescence (TRPL) spectra were excited at a wavelength of 375 nm and collected by a fiber coupled to a HORIBA iHR320 spectrometer connected with a Hamamatsu Universal Streak Camera C10910 at the National Synchrotron Radiation Research Center (NSRRC) of TPS 23A. The morphology of perovskite nanostructures were revealed by scanning electron microscope (SEM, JEOL JSM-6500F, Tunghai university, Taichung city, Taiwan). The crystal structures of perovskite NCs were determined with a powder X-ray diffractometer (Rigaku Miniflex 600, Tunghai university, Taichung city, Taiwan). In situ GISAXS was conducted at the 23A SWAXS beamline of the Taiwan Light Source of the National Synchrotron Radiation Research Center, Hsinchu. Microscopic PL mappings were taken with a confocal spectroscopic system (South Port, Jade Mat, Taipei, Taiwan), where a 532 nm continuous-wave laser was used as the excitation source.

## Figures and Tables

**Table 1:** Photovoltaic characteristics of 2D RP PSCs with and without urea treatment under AM 1.5G illumination (100 mW/cm^2^).

	Jsc (mA)	Voc (mV)	FF (%)	h (%)
With Urea	11.4	1103.6	62.82	7.9
Without Urea	1.4	771	59.08	0.64

## Data Availability

Not applicable.

## Supplementary Materials

The following supporting information can be downloaded at: https://www.mdpi.com/article/10.3390/nano12111816/s1. Figure S1: The schematic illustration of chemical structure of RP 2D perovskite materials; Figure S2: XRD features of the pristine film and urea treated film prepared from a precursor solution of (a) (BA)_2_Pb_1_I_4_ and (b) (BA)_2_(MA)_1_Pb_2_I_7_; Figure S3: The photo conversion efficiency (PCE) of the device were tracked over 240 hours under ambient condition; Figure S4: J–V curves of 2D RP PSCs; Figure S5: 2D intensity mapping of the PL spectra for a 5 µm × 5 µm area of (a) the pristine film and (b) the urea treated film from a precursor of urea treated (BA)_2_(MA)_1_Pb_2_I_7_ solution; Table S1: The PL life time was calculated from TRPL spectra; Table S2: The calculation of crystallite size of 2D perovskite materials with/without urea treatment.
