# Supplementary material for: Accelerated Formation of 2D Ruddlesden—Popper Perovskite Thin Films by Lewis Bases for High Efficiency Solar Cell Applications
Source: Nanomaterials (Basel). 2022 May 26;12(11):1816. doi: 10.3390/nano12111816 (PMC9182189; doi:10.3390/nano12111816)
Supplement: Supplementary file 1 [file nanomaterials-12-01816-s001.zip › nanomaterials-1711022-supplementary.pdf]

Supporting information

Accelerated Formation of 2D Ruddlesden–Popper Perovskite Thin Films by Lewis Bases for High Efficiency Solar Cell Applications

Swathi M. Gowdru [1,†], Jou-Chun Lin [1,†], Szu-Tan Wang [1,†], Yi-Chia Chen [1], Kuan-Chang Wu [1], Cheng-Nan Jiang [1], Yu-Dian Chen [1], Shao-Sian Li [2,*], Yuan Jay Chang [1] and Di-Yan Wang [1,*]

[1] Department of Chemistry, Tunghai University, Taichung 40704, Taiwan; swathimb3@gmail.com (S.M.G.); jouchun1908@thu.edu.tw (J.-C.L.); ybbis1124@yahoo.com.tw (S.-T.W.); yichia899@gmail.com (Y.-C.C.); leo19981009leo@gmail.com (K.-C.W.); jiangjengnan@gmail.com (C.-N.J.); 510087d@gmail.com (Y.-D.C.); jaychang@thu.edu.tw (Y.J.C.)

[2] Department of Materials and Mineral Resources Engineering, Institute of Materials Science and Engineering, National Taipei University of Technology, Taipei 10608, Taiwan

* Correspondence: ssli@ntut.edu.tw (S.-S.L.); diyanwang@thu.edu.tw (D.-Y.W.)

† These authors contributed equally to this work.

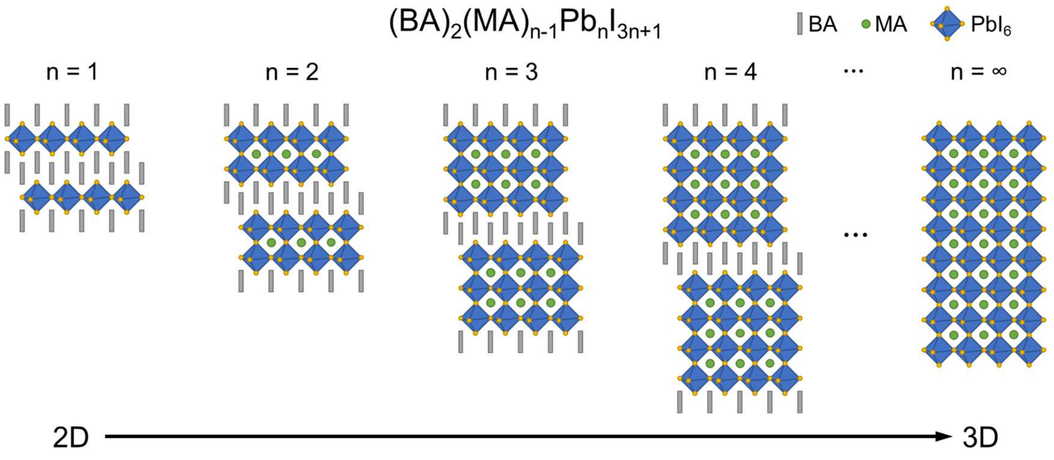

Figure S1. The schematic illustration of chemical structure of $(BA)_2(MA)_{n-1}Pb_nI_{3n+1}$ 2D perovskite unit cell with different n value.

The TRPL spectra of pristine and urea treated films were fitted by a bi-exponential decay function as shown below:

$$A(t) = A_0 + A_1 e^{(-\frac{t}{\tau_1})} + A_2 e^{(-\frac{t}{\tau_2})} \quad (1)$$

Where A_0 is a constant for the baseline offset, A_1 and A_2 are the amplitudes of the TRPL decay, τ_1 is the fast decay time responsible for interface (trap-assisted) recombination, and τ_2 is the slow decay time responsible for bulk (carrier radiative) recombination. The average lifetimes (τ_{ave}) were estimated with the function as:

$$\tau_{ave} = \frac{A_1 {\tau_1}^2 + A_2 {\tau_2}^2}{A_1 \tau_1 + A_2 \tau_2} \quad (2)$$

The obtained results of TRPL data were listed in the table below. The results showed the obtained pristine films exhibit a short decay time (τ1) of 2.32 ns accounting for 88.5%, a long decay time (τ2) of 6.38 ns accounting for 11.5%, and average lifetime (τave) of 3.39 ns. In comparison, the TRPL fitting results of urea teated films show τ1 of 1.47 ns accounting for 94.4%, τ2 of 4.68 ns accounting for 5.56%, and average lifetime (τave) of 1.98 ns. The increament of interface recombination indicated that urea treatment can improving the film quality.

Table S1. The PL life time was calculated from TRPL spectra of pristine and urea treated films which was fitted by a bi-exponential decay function.

	λ_{Em} [nm]	A_1 [%]	τ_1 [ns]	A_2 [%]	τ_2 [ns]	τ_{ave} [ns]
with urea	675	94.4	1.47	5.56	4.68	1.98
without urea	725	88.5	2.32	11.5	6.38	3.39

Table S2. The calculation of crystallite size of 2D perovskite materials with/without urea treatment by the Scherrer equation from XRD spectra in Figure 2 (a).

	Plane	2 Theta (deg.)	d-spacing (Å)	Intensity ratio of (111)/(202)	Crystallite size (nm)
**Without urea**	(111)	14.1	6.35	0.16	39.4
	(202)	28.5	3.12		
**With urea**	(111)	14.1	6.35	1.11	42.1
	(202)	28.5	3.12		

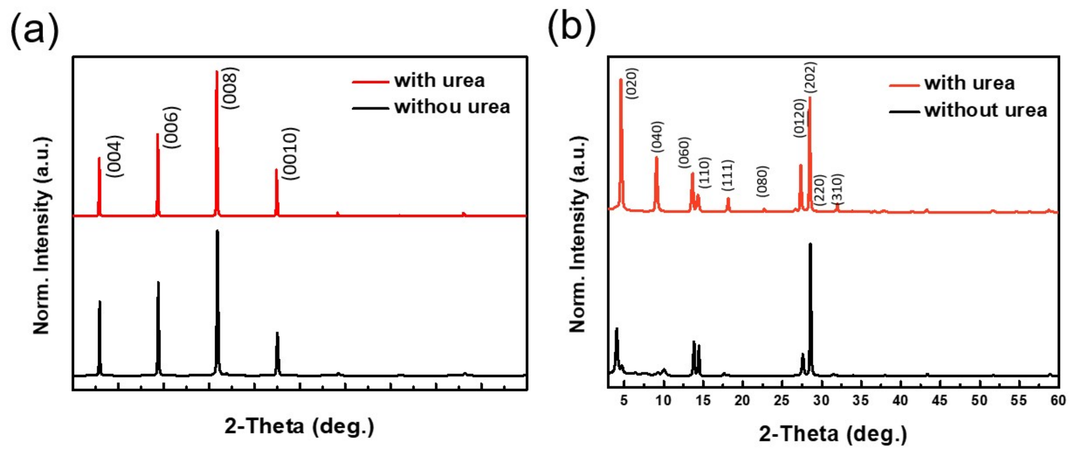

Figure S2. XRD features of the pristine film and urea treated film prepared from a precursor solution of (a) $(BA)_2Pb_1I_4$ and (b) $(BA)_2(MA)_1Pb_2I_7$.

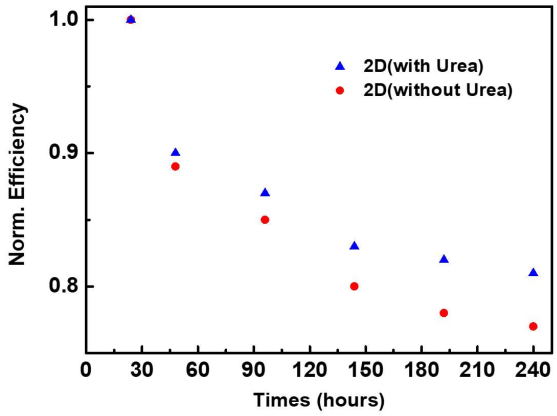

Figure S3. The photo conversion efficiency (PCE) of the device were tracked over 240 hours under ambient condition (the temperature of ~25 ◦C and humidity of ~60 RH%).

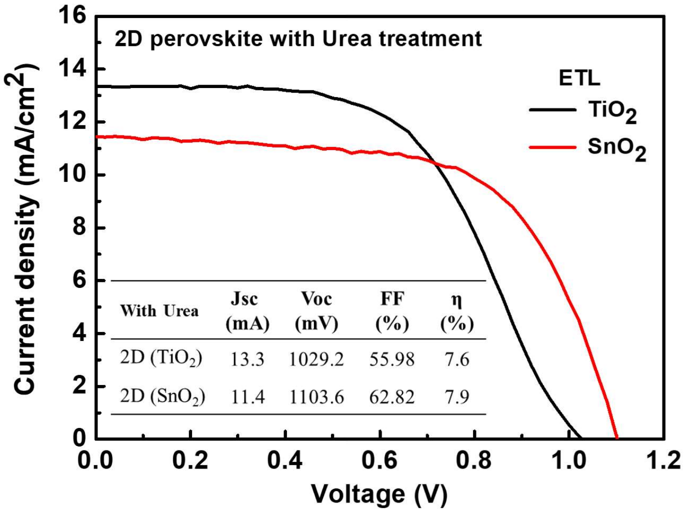

Figure S4. J–V curves of 2D RP PSCs with using TiO_2 and SnO_2 as ETL under simulated AM 1.5G illumination (100 mW/cm^2).

To find out the effect of ETL on the PCE of 2D perovskite solar cell device, TiO_2 film was deposited on FTO in comparison with SnO_2 on ITO. Here, SnO_2 was used as ETL over its counter part of TiO_2 due to the superior physical and chemical properties such as high electron mobility, high conductivity and less photo-activity to provide better stability under light illumination. However, the conduction band minimum (CBM) of SnO_2 is slightly deeper than CBM of TiO_2, which might reduce the value of Voc when 2D ruddleson popper perovskite was used as light absorber. Chemical modification of CPTA was applied onto SnO_2 to create dipole between ETL and perovskite layer to enlarge the built-in potential at SnO_2/CPTA interface. This resulted in a better energetic cascade structure for photo-generated electrons transferring to the ITO and therefore sustained a better electron potential and higher Voc as shown in **Figure S4**. The modification also promoted the ability of electron transfer and suppress recombination, leaded to a higher solar cell performance. By contrast, there is only physical contact between TiO_2 and 2D perovskite layer (no interfacial dipole), significant charge recombination could not be prohibited and giving to a lower Voc. However, the rougher surface of TiO_2 ETL provide higher interface area for charge separation than SnO_2 ETL, and increase the photocurrent (Isc).

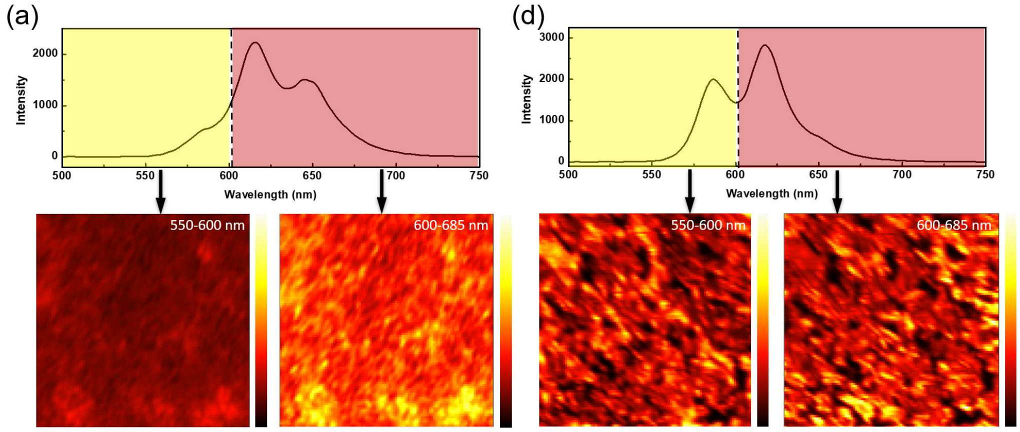

Figure S5. 2D RP perovskite film with mixed phases (n=1, 2 and 3) was prepared by a precursor of urea treated $(BA)_2(MA)_1Pb_2I_7$ solution with using spin coating process. 2D intensity mapping of the PL spectra for a 5 μm × 5 μm area of (a) the pristine film and (b) the urea treated film. The two wavelength ranges (one is 550 nm to 600 nm and the other is 600 nm to 685 nm) of PL mapping were selected. According to the PL mapping, we found that with urea treatment, the 2D RP film with mixed n values (including n=1, 2 and 3) did form successfully over a large area compared to the pristine films, which tended to form 3D phases.
